# Predicting Antibiotic Resistance in *Listeria monocytogenes* from Food and Food-Processing Environments Using Next-Generation Sequencing: A Systematic Review

**DOI:** 10.3390/ijms262010112

**Published:** 2025-10-17

**Authors:** Patryk Wiśniewski, Patryk Adamski, Miłosz Trymers, Wioleta Chajęcka-Wierzchowska, Anna Zadernowska

**Affiliations:** Department of Food Microbiology, Meat Technology and Chemistry, Faculty of Food Science, University of Warmia and Mazury, Plac Cieszyński 1, 10-726 Olsztyn, Poland; patryk.adamski@uwm.edu.pl (P.A.); milosz.trymers@uwm.edu.pl (M.T.); wioleta.chajecka@uwm.edu.pl (W.C.-W.); anna.zadernowska@uwm.edu.pl (A.Z.)

**Keywords:** NGS, whole-genome sequencing, resistance determinants, antimicrobial resistance, genotypic–phenotypic correlation

## Abstract

*Listeria monocytogenes* is a ubiquitous foodborne pathogen whose occurrence in food and food-processing environments raises public-health concerns, particularly when isolates carry antimicrobial-resistance determinants. Next-generation sequencing (NGS) is increasingly used to detect resistance genes and to predict phenotypic resistance. Following the Preferred Reporting Items for Systematic Reviews and Meta-Analyses (PRISMA 2020) guidelines, PubMed, Web of Science, and Scopus were searched for original articles (2015–2024) that used second- and/or third-generation sequencing to characterize antibiotic resistance in *L. monocytogenes* from food and food-processing environments. After deduplication and screening, 58 studies were included from an initial 418 records. NGS reliably detected a set of recurrent resistance determinants across diverse sample types and geographies. The *fosX* locus (intrinsic fosfomycin-related marker) was effectively ubiquitous across studies, while acquired determinants were variably distributed: *lin* (35/58 studies, 60.34%), *norB* (33/58, 56.90%), and tetracycline genes overall in 20/58 (34.48%) with *tetM* as the most common (11/58, 18.97%). Reported concordance between the genotypes and phenotypes for acquired resistance was very high (>99% for most agents), with notable exceptions (e.g., ciprofloxacin and some fosfomycin cases). Common analysis pipelines and databases included ResFinder, CARD, BIGSdb-Lm, ABRicate, and ARIBA; most sequencing used Illumina short reads, with an increasing use of long-read or hybrid approaches. NGS is a powerful surveillance tool for detecting resistance determinants and for source-tracking, but its predictive value depends on integration with phenotypic testing, standardized reporting, and comprehensive, curated databases. Key gaps include inconsistent phenotype reporting, variable database coverage, and limited assessment of gene expression/regulatory effects.

## 1. Introduction

*Listeria monocytogenes* is a highly ubiquitous, multi-resistant foodborne pathogen found in soil, water, vegetation, and animal reservoirs [1]. This pathogen can grow over a wide range of temperatures and environmental pH and persist in food-processing environments, making it an extremely significant challenge for food safety [2]. *L. monocytogenes* is responsible for causing listeriosis. Human listeriosis has high rates of hospitalization and mortality, especially in vulnerable groups, and is on the rise in Europe despite efforts to control it [3,4]. Listeriosis therapy can be complicated because *L. monocytogenes* is an intracellular pathogen, so some antibiotics show efficacy in vitro, but in vivo, they only have a bacteriostatic effect [5]. In recent years, concerns about antibiotic-resistant *L. monocytogenes* in food and production environments have increased [2,6,7]. For example, a study by Rippa et al. (2024) [2] found alarmingly high levels of resistance—87.36% (n = 235) of 269 *L. monocytogenes* strains isolated from food and food-production environments were multidrug resistant (MDR). Similarly, in a Nigerian study by Kayode and Okoh (2023) [6], of 194 isolates from ready-to-eat (RTE) foods, >50% of strains resistant to sulfamethoxazole or trimethoprim and >40% resistant to amoxicillin, penicillin, or erythromycin were observed. These findings indicate that *L. monocytogenes* carrying resistance to clinically relevant antibiotics may be present in a variety of food sources (meat, dairy, seafood/vegetable RTE foods, etc.), raising public health concerns.

Importantly, *L. monocytogenes* has known intrinsic resistance traits that shape its antibiogram. The literature reports emerging acquired resistance in *L. monocytogenes*. A recent genomic study of 5339 French isolates (2012–2019) showed that only 2.23% contained acquired antimicrobial resistance (AMR) genes, and these were much more common in food-derived isolates (3.74%) than in clinical isolates (0.98%) [8]. Although most *L*. *monocytogenes* remain susceptible to standard treatment, the food chain contains a growing fraction of strains with genetic resistance traits [8].

The standard approach for antimicrobial resistance detection testing in microorganisms, including *L. monocytogenes*, is phenotypic testing. Typical methods include the following: the disk-diffusion method (Kirby–Bauer method) or the broth microdilution method to determine minimum inhibitory concentrations (MICs) against a panel of antibiotics [7]. Phenotypic tests characterize isolates as the following: “susceptible, standard dosing regimen (S)”, “susceptible, increased exposure (I)”, and “resistant (R)” for a specific antimicrobial agent [9]. These methods, while necessary for antimicrobial resistance diagnosis, have several drawbacks—they can be lengthy (requiring 1–2 days of culture), they may not reveal underlying genetic mechanisms, and their precision depends on standardized breakpoints that can miss low levels or newly occurring resistance [10]. They provide only a superficial picture of resistance and do not detect emerging resistance genotypes. Resistant isolates are identified in these methods by their growth, but the specific genes involved in encoding resistance to specific agents or mutations in these genes remain unknown [11].

In recent years, whole-genome sequencing (WGS), a next-generation sequencing (NGS) method, has become an increasingly important tool in the study of antibiotic resistance, including in *L. monocytogenes* [12]. WGS enables the high-resolution detection of resistance genes and mutations, including both chromosomal and mobile genetic elements, offering insights beyond the reach of traditional phenotypic methods [13]. Beyond identifying resistance genes, WGS allows researchers to assess their genomic context (e.g., plasmids and transposons), assign isolates to specific sequence types (STs) or clonal complexes (CCs), and trace their transmission in food-production chains [13].

This literature review aims to summarize original research published in the three main databases (PubMed, Web of Science, and Scopus) that applied whole-genome next-generation sequencing to investigate antibiotic resistance in *L. monocytogenes* isolated from food and food-processing environments.

## 2. Methods

The criteria following the Preferred Reporting Items for Systematic Reviews and Meta-Analyses (PRISMA 2020) were used to prepare a systematic review of original scientific articles.

The analysis involved searching three open-access research databases: PubMed, Web of Science, and Scopus (last search: 18 March 2025). These databases were used to find research articles specifically focused on using next-generation sequencing as a tool for predicting antibiotic resistance, by analyzing the presence of antibiotic resistance genes in *L. monocytogenes* isolates obtained from food in its production environment.

Inclusion criteria for the database search were (a) original research papers published between 2015 and 2024; (b) characterization of *L. monocytogenes* isolates from food and the food-production environment; and (c) the use of second- and/or third-generation sequencing technology.

The following criteria were used as exclusion criteria: (1) no/other source of strains than food and food-processing environment; (2) other than the original research article; (3) no analysis of *L. monocytogenes* strains; (4) no use of NGS for the analysis of *L. monocytogenes*; (5) different context for research than antibiotic resistance; (6) no analysis of antibiotic resistance genes; and (7) another reason. Search terms “*Listeria monocytogenes*” OR “*L. monocytogenes*” AND “next generation sequencing” AND “resistance” OR “*Listeria monocytogenes*” OR “*L. monocytogenes*” AND “whole-genome sequencing” AND “resistance”. After removing duplicate records from the databases, 418 results were obtained, of which 58 met the inclusion criteria. The database search and literature analysis identified key original research studies focusing on the use of next-generation sequencing in predicting antibiotic resistance for *L. monocytogenes* strains isolated from food and food-production environments (Table 1) [8,12,14,15,16,17,18,19,20,21,22,23,24,25,26,27,28,29,30,31,32,33,34,35,36,37,38,39,40,41,42,43,44,45,46,47,48,49,50,51,52,53,54,55,56,57,58,59,60,61,62,63,64,65,66,67,68,69]. Data items: country, year of sample collection, sample type (food/processing environment), number of isolates, sequencing platform, bioinformatics pipeline, phenotypic resistance testing, and resistance genes detected (Table 1 and Table S1 and Figure S1).

**Figure 1:**
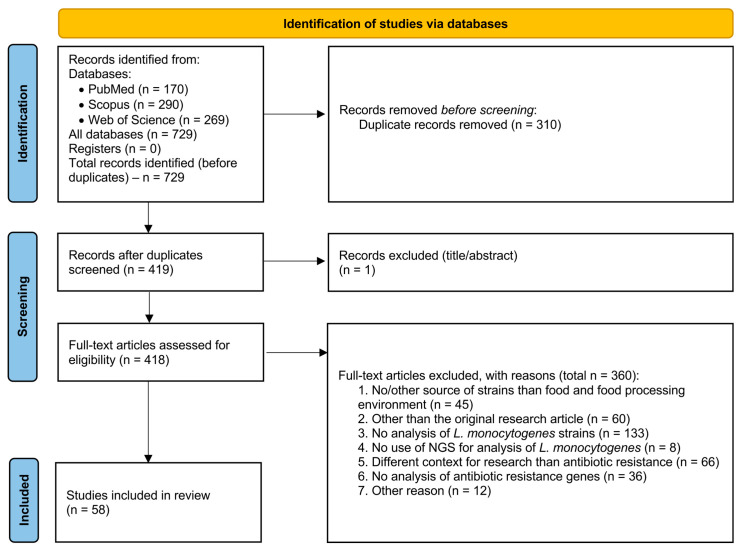
A summary of the selection process for all publications reviewed for the study.

The entire selection process of the publications analyzed for this study is shown in Figure 1 and summarized in Table S1. Full search strategies for each database are provided in Supplementary Table S2. Duplicates were removed by P.W. and checked by P.A. and M.T. Data extraction was performed independently by P.W., P.A., and M.T. Extracted items were verified by A.Z. and W.C.-W. Discrepancies were resolved through discussion. Due to heterogeneity in study designs, sequencing methods, and reported outcomes, a meta-analysis was not performed. Results are synthesized narratively and presented in tables summarizing study characteristics and findings.

The authors (P.W., P.A., and M.T.) independently reviewed the titles and abstracts of the retrieved articles, while any disagreements were resolved through discussion with the next authors (A.Z. and W.C.-W.).

## 3. Results and Discussion

### 3.1. Profiling Resistance Genes as Phenotypic Predictors in L. monocytogenes Isolated from Food and Food-Production Environments

In recent years, there has been an increasing focus on the genetic markers of antibiotic resistance as potential predictors of phenotypic resistance. Among *L. monocytogenes*, there are both innate resistance mechanisms (such as natural resistance to cephalosporins or glycopeptides) and acquired resistance genes. Scientific studies have shown that the presence of specific genes strongly correlates with the phenotypic resistance profiles observed. It has been demonstrated that the phenotype of acquired antibiotic resistance in *L. monocytogenes* can be predicted from genome sequence analysis with an accuracy of over 99%. This indicates that strains carrying characteristic genes (e.g., *tetM*, *lnuG*, *ermB*, *mphB*, *fexA*, etc.) typically exhibit resistance to their respective antibiotics. Frequently transferred on plasmids or other mobile genetic elements, the *tet* and *erm* genes have also been documented in numerous studies of food-derived *L. monocytogenes.*

Due to the diverse antibiotic resistance profiles of *L. monocytogenes* strains isolated from food and food-production environments, molecular genotypic profiling is becoming a key part of microbial threat monitoring. Analyzing the presence of resistance genes in bacterial populations enables the early detection of rising resistance trends and the identification of potentially dangerous strains. Gene profiling can serve as a rapid marker of potential phenotypic resistance even before comprehensive phenotyping tests are completed. Whole-genome sequencing offers a complete view of a strain’s genome, allowing for the detection of all known resistance determinants, establishing strain relatedness, and tracing sources of contamination in the food chain. WGS analyses have proven useful in routine surveillance and epidemiological studies—enabling precise typing and source tracking of listeriosis outbreaks, as well as the identification of new vectors for resistance gene transmission in the production environment.

**Figure 2:**
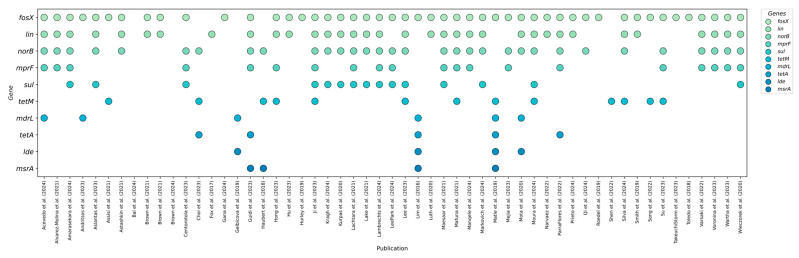
The most frequently determined antibiotic resistance genes in *L. monocytogenes* from food and food-production environments that were analyzed in the studies [8,12,14,15,16,17,18,19,20,21,22,23,24,25,26,27,28,29,30,31,32,33,34,35,36,37,38,39,40,41,42,43,44,45,46,47,48,49,50,51,52,53,54,55,56,57,58,59,60,61,62,63,64,65,66,67,68,69].

To highlight the benefits of next-generation sequencing in profiling antibiotic resistance genes, fifteen of the most common determinants were selected based on a literature review following established criteria. Figure 2 provides a summary of these genes, confirmed by numerous studies on *L. monocytogenes* strains isolated from food and production environments.

### 3.2. Prevalent Resistance Genes and Their Predictive Value

Based on a literature search following previously established criteria, the fifteen resistance genes most frequently detected in *L. monocytogenes* were identified through next-generation sequencing. Sequencing not only enables the simultaneous detection of all known genetic determinants of antibiotic resistance but also allows for the assessment of their prevalence in populations isolated from food and food-production environments. The articles shown in Figure 2 report the prevalence of genes such as *fosX*, *lin*, *norB*, *mprF*, *sul*, *tetM*, *mdrL*, *tetA*, *aacA4*, *tet*, *msrA*, *lde*, *tetS*, *fexA*, and *mepA*, which exhibited the highest detection rates in various studies. By compiling the results from these studies, we can identify which resistance mechanisms are most common in the food sector. An overview of the genetic profiles, including the number and percentage of strains with each gene in each study, provides a foundation for further risk analysis and the development of predictive models for phenotypic resistance based on NGS data.

**Figure 3:**
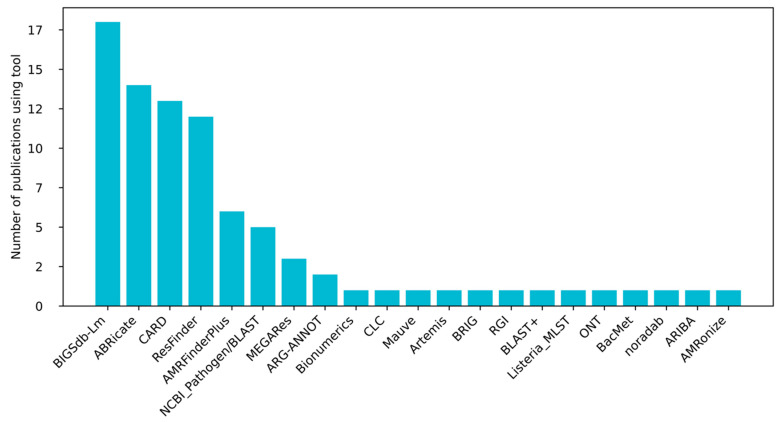
The use of bioinformatic tools in the prediction of resistance genes in the analyzed studies.

Methodologically (Figure 3), detection relied on Illumina platforms (MiSeq, NextSeq, HiSeq, and NovaSeq) and was analyzed using tools such as ResFinder (Center for Genomic Epidemiology DTU; https://genepi.food.dtu.dk/resfinder (accessed on 21 July 2025)), Comprehensive Antibiotic Resistance Database/Resistance Gene Identifier (CARD/RGI; https://card.mcmaster.ca/, https://card.mcmaster.ca/analyze/rgi (accessed on 21 July 2025)), Bacterial Isolate Genome Sequence Database—*Listeria monocytogenes* (BIGSdb-Lm; https://bigsdb.pasteur.fr/listeria/ (accessed on 21 July 2025)), ABRicate (GitHub; https://github.com/tseemann/abricate (accessed on 21 July 2025)), or ARIBA (Antimicrobial Resistance Identification By Assembly, GitHub; https://github.com/sanger-pathogens/ariba (accessed on 21 July 2025)). Despite minor variations in read length and assembly pipelines, every study reported robust identification of antibiotic resistance genes, underscoring their stability across sequencing technologies and bioinformatic workflows.

The detection of antibiotic resistance genes in WGS studies typically relies on short-read Illumina sequencing, though long-read approaches are increasingly used. In most studies, *L. monocytogenes* genomes were generated on the Illumina MiSeq, HiSeq, NextSeq, NovaSeq, X10, and Nanopore platforms (150 bp paired-end reads, 250 bp paired-end reads, and 300 bp paired-end reads). Illumina reads provide high per-base accuracy (<0.1% error) but produce fragmented assemblies due to short read length [70]. In contrast, Oxford Nanopore long reads (10 kb+) can span repetitive regions and yield complete genomes, albeit with higher error rates. In practice, hybrid assembly (combining short and long reads) can recover full chromosomes and mobile elements. Regardless of platform, all studies report robust detection of AMR genes once the genome is assembled or even directly from reads [71].

**Figure 4:**
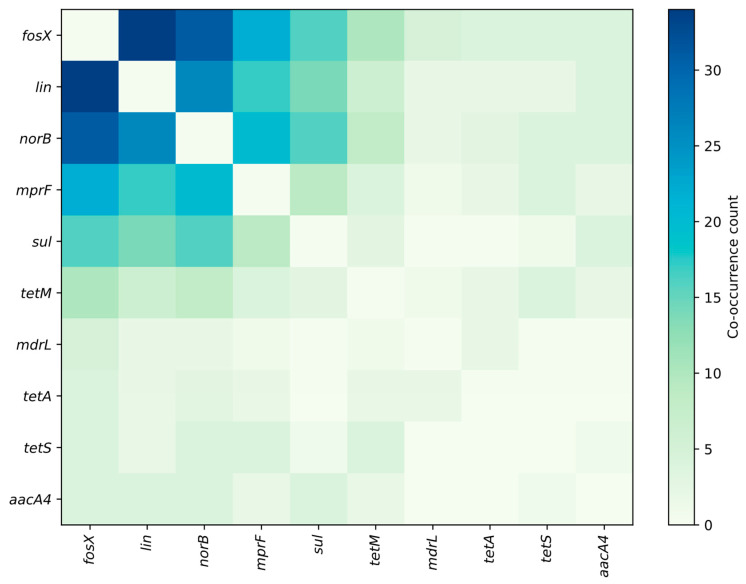
Frequency matrices for the detection and co-occurrence of resistance genes (top 10 genes).

**Figure 5:**
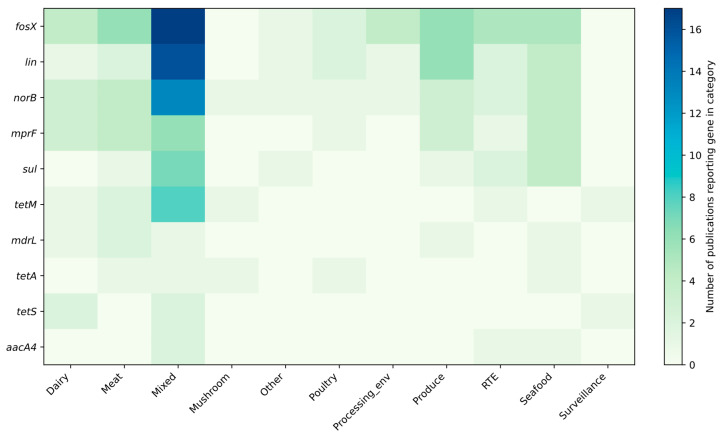
Frequency of reporting resistance genes in individual sample categories in the analyzed studies (top 10 genes).

**Figure 6:**
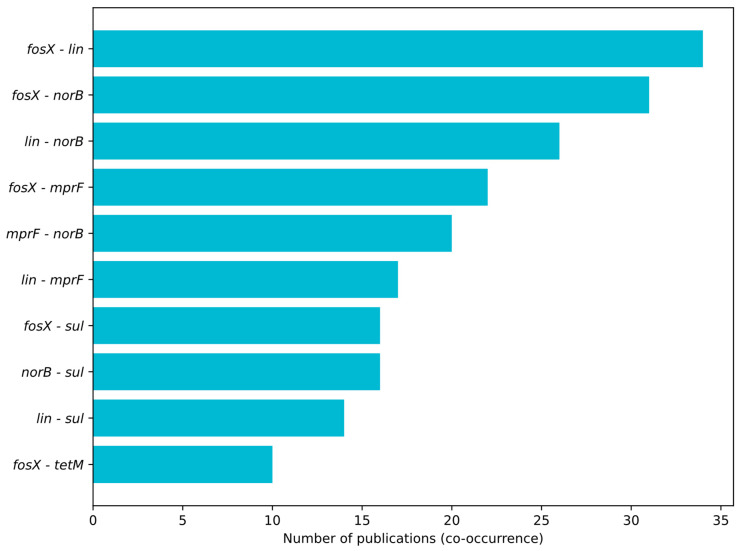
Top 10 gene pairs with the highest co-occurrence—number of publications.

Figure 4, Figure 5 and Figure 6 summarize the frequency and co-occurrence patterns of the top ten resistance genes across the surveyed publications. The co-occurrence matrix (Figure 4) displays the number of publications in which gene pairs were detected together; the strongest and most frequently reported pairings are *fosX–lin*, *fosX–norB*, and *lin–norB*. The gene-sample heatmap (Figure 5) shows the distribution of detections across sample categories and indicates that the greatest number of reports for *fosX*, *lin*, and *norB* originate from meat-related samples, while other sample types yield fewer but still notable detections. The bar chart of the top ten co-occurring gene pairs (Figure 6) ranks pairs by the number of publications reporting their simultaneous presence, with *fosX–lin* at the top, followed by *fosX–norB*, *lin–norB*, and additional frequent combinations involving *mprF* and *sul*. Collectively, these figures present a clear, publication-based picture of which resistance determinants and gene combinations are most commonly reported in *L. monocytogenes* from food and production environments, supporting targeted surveillance and comparative analyses of resistance prevalence.

#### 3.2.1. Fosfomycin Resistance

The *fosX* gene is the most frequently detected resistance gene among scientific studies analyzing the genome sequencing of *L. monocytogenes* strains isolated from food and food-production environments [8,12,14,15,16,17,18,19,20,21,22,23,26,28,30,31,32,33,34,35,36,37,38,40,41,42,43,44,45,46,47,48,49,51,52,53,54,55,57,58,59,60,61,62,63,64,65,66,68,69,72].

Across 50 independent studies spanning five continents, *fosX* was detected in every surveyed population—whether in traditional dairy products [17,43], ready-to-eat (RTE) meat [23,35], poultry [28], beef production chains [12,26], seafood [36,68], fresh produce [16,49], or environmental swabs from food-processing facilities [36,65].

The *fosX* gene (*lmo1702* in strain EGD-e) encodes a Mn^2+^-dependent epoxide hydrolase that inactivates fosfomycin. It is part of the *L. monocytogenes’* core genome and confers intrinsic resistance to fosfomycin [73,74]. Genomic analyses confirm that *fosX* is nearly ubiquitous across *L. monocytogenes* (and related *Listeria* sensu stricto) strains [74]. FosX-mediated resistance is encoded in the listerial core genome of all examined pathogenic and non-pathogenic *Listeria*, as demonstrated by Manqele et al. (2024) [12] and Parra-Flores et al. (2022) [51], who detected *fosX* in 100% of isolates from beef and ready-to-eat foods, respectively. Even in highly diverse sample sets—including 2431 isolates across Europe [8] and 322 Chinese isolates from meat, aquatic products, and RTE foods [33]—*fosX* prevalence consistently approached 100 %. No difference in *fosX* prevalence has been observed between food-derived and clinical isolates (both ~100%), underscoring its status as a core, chromosomal gene.

Standard antimicrobial resistance gene databases are used to search for *fosX* genes in *L. monocytogenes* sequences. Commonly used tools such as ResFinder (from CGE), the CARD database, and BIGSdb-Lm routinely include *fosX* for *Listeria*. Specifically, ResFinder identified *fosX* as the sole fosfomycin resistance gene in isolates from food and food-processing environments. Likewise, BIGSdb-Lm (Pasteur Institute’s *Listeria* scheme) annotates *fosX* as an intrinsic resistance locus. In a Polish study, BIGSdb-Lm scanning flagged *fosX* in all 91 isolates [36]. The study by Manqele et al. (2024) [12] screened their Illumina assemblies with ABRicate using ResFinder, CARD, and the NCBI AMR database; all three tools recovered *fosX* in every genome. Thus, whether using BLAST/ABRicate on ResFinder/CARD or querying the BIGSdb-Lm allele database, *fosX* is consistently recovered.

Although the *fosX* gene is present in almost 100% of *L. monocytogenes* isolates, data on phenotypic resistance to fosfomycin remain limited and inconclusive. Genotype–phenotype correlations are not well documented—*fosX* is common even when the strain is phenotypically susceptible to fosfomycin [8,74,75]. It is worth including this in the research analyses of phenotypic resistance to fosfomycin in strains containing this gene, as well as studies on changes in gene expression under the influence of various stress factors. However, the presence of *fosX* is considered an important marker of core resistance, which may be relevant for predictive analyses (genotype–phenotype) and the assessment of the natural tolerance range of the species [76]. Moreover, the gene *fosX* and phenotypic resistance to fosfomycin were detected [65,68]. Scortti et al. (2018) [74] described that, despite *fosX* gene expression, *L. monocytogenes* isolates were susceptible to the antibiotic fosfomycin due to epistasis, but only during infection.

#### 3.2.2. Lincosamides Resistance

The resistance of *L. monocytogenes* to lincosamides is based, among other things, on mechanisms that modify the target site of the antibiotic, i.e., the ribosome. The major gene responsible for lincosamide resistance is the *lin* gene, which encodes the enzyme nucleotidyl transferase (LinA), which inactivates the antibiotic by adenylation of its molecule. The *lin* gene is responsible for the direct chemical modification of the structure of lincosamides, resulting in their neutralization [77,78,79]. In the analyzed studies, the *lin* gene was detected in 35 cases (35/58; 60.34%) [8,12,14,15,16,18,20,28,30,31,33,34,35,36,37,38,40,42,43,44,45,48,49,51,52,53,57,58,61,63,64,65,66,68]. Among the bioinformatic tools most commonly used for its detection, BIGSdb-Lm dominated and was used in 14 publications (14/35; 40.00%) [8,16,18,20,28,33,34,35,36,37,38,43,45,66]. It was followed by ABRicate, used in nine cases (9/35; 25.71%) [14,15,42,44,48,49,58,65,66], and ResFinder, present in six studies (7/35; 20.00%) [12,15,38,49,57,63]. The *lin* gene has been detected, among others, in *L. monocytogenes* isolates from fish and seafood [36,68], meat and meat products [12,35,44,45,47], vegetables and fruits [16,37,49,51], spices and plant products [31], food-production environments [14,34,36,65], poultry [28], and ready-to-eat products [61].

The *erm* (erythromycin r-methyltransferase) family of genes, particularly *ermB*, which encodes ribosomal methylases that modify 23S rRNA, play a key role here. This change prevents the antibiotic from binding to the ribosome, leading to MLSB (macrolides-lincosamides-streptogramins B) resistance [77,80,81]. The fact that the *ermB* gene is detected, among others, in *L. monocytogenes* isolates from the food-processing environment suggests a horizontal transfer of resistance, especially by transposons such as Tn917 or Tn1545 class elements [82]. The results of our analysis indicated that the *ermB* gene was detected in two cases (2/58; 3.45%). Both publications used the CARD program (2/2; 100.00%) as a tool for identifying resistance genes [8,60]. It has also been shown that the presence of *ermB* may correlate with the *tetM* gene, indicating co-selection of resistance to tetracyclines and lincosamides [83].

The *vga(G)* gene is another gene that determines phenotypic resistance to lincosamides [84]. In a study conducted by Gana et al. (2024) [26], the *vga(G)* gene, which encodes an ABC-F family ribosome protection protein, was detected in all 60 *L. monocytogenes* isolates analyzed. These results are consistent with observations from South Africa, where some antibiotics are available without a prescription and are used in animal husbandry for both therapeutic purposes and as growth promoters [26]. Similarly, Lee and Park (2024) [39] report the presence of the *vga(G)* gene in *L. monocytogenes* isolates, confirming the widespread distribution of lincosamide resistance determinants.

The next gene responsible for encoding resistance to lincosamides is *lunG*, discovered by Moura et al. (2024) [8]. This gene encodes the enzyme nucleotidyl transferase, which is responsible for inactivating antibiotics from this group through their adenylation. This process leads to a reduction in the therapeutic efficacy of lincosamides, which poses a significant threat from the point of view of clinical therapy [85]. Importantly, the same study also demonstrated the presence of other genes associated with antibiotic resistance, such as *aphA*, *dfrD*, *ermB*, *mphB*, and *pbp*, which encodes the mechanisms determining resistance to aminoglycosides, trimethoprim, macrolides, and β-lactams, among others. The presence of such a wide repertoire of resistance determinants indicates the high potential of the studied strains to inhibit the action of many classes of antibiotics, which significantly hinders their control and emphasizes the need to monitor the spread of these genes in clinical and non-clinical environments [8].

#### 3.2.3. Resistance to Fluoroquinolones

The *norB* gene is responsible for encoding a chromosomally located multidrug efflux pump, classified within the Major Facilitator Superfamily (MFS), and is associated with resistance to fluoroquinolones, particularly conferring resistance to both hydrophobic quinolones, such as sparfloxacin and moxifloxacin, and hydrophilic quinolones, including norfloxacin and ciprofloxacin [86,87].

In the analyzed studies, the *norB* gene was detected in 33 cases (33/58; 56.90%) [8,12,14,15,16,18,20,23,24,28,29,33,34,35,36,37,38,39,40,43,44,45,47,48,51,54,57,60,63,64,65,66,68]. Among the bioinformatic tools most commonly used for its detection, BIGSdb-Lm dominated, used in 15 publications (15/33; 45.45%) [8,16,18,20,28,33,34,35,36,37,38,43,64,66,88], next, CARD (10/33; 30.30%) [12,15,51,54,57,60,63,64,65,68]. Following were ABRicate, used in seven cases (7/33; 21.21%) [14,15,44,48,63,65,66] and ResFinder used in six works (6/33; 18.19%) [12,15,38,48,57,63]. The following were used in isolated cases: MEGARes database [83], AMRFinderPlus [40], *Listeria* MLST database, AMRFinderPlus [39], *Listeria* MLST database [40], ARG-ANNOT [65]. The *norB* gene was detected, among others, in *L. monocytogenes* isolates from slaughterhouses, meat, and meat products [15,20,28,35,44], fish and seafood [20,33,66,68], vegetables and fruit [16,34,37,51], and food-production environments [34] as well as ready-to-eat products [8].

Other genes responsible for fluoroquinolone resistance are as follows: *GYRA_23* [29,47], *parC* [28], and *fepA* [48].

The *GYRA_23* gene, the presence of which has been linked to fluoroquinolone resistance, was detected in strains isolated from dairy products in studies conducted by Mejía et al. (2023) [47] and Haubert et al. (2018) [29]. However, analysis using different databases revealed discrepancies in its identification—the gene was not shown in the CARD database, while it was identified by MEGARes [47].

A study by Guidi et al. (2023) [28] demonstrated the presence of genes determining quinolone resistance in selected populations of *L. monocytogenes*; in addition to the common AMR genes, the authors observed the presence of the *parC* gene (encoding the C subunit of topoisomerase II), responsible for quinolone resistance, in all isolates studied.

In contrast, Mota et al. (2020) [48] identified the *fepA* gene as an important factor in *L. monocytogenes’* resistance to fluoroquinolones. The mechanism is mainly related to the activity of pumps that pump the antibiotic out of the cell—the overexpression of *fepA* enables efficient removal of the drug, reducing its intracellular concentration [89]. The genomic analyses also searched for sequences in the NCBI BLAST database, which allowed us to identify additional genes responsible for resistance, including *fepA*, which—despite their description in the literature—are not always listed in commonly used databases such as ResFinder, CARD, NCBI AMRFinderPlus, or MEGARes.

#### 3.2.4. Antimicrobial Peptides Resistance

The *mprF* gene encodes lysylphosphatidylglycerol synthetase, an enzyme that uses phosphatidylglycerol as its substrate. This reaction leads to the formation of lysyl-phosphatidylglycerol and diphosphatidylglycerol, both of which are essential structural components of the cell wall. As a result, bacteria exhibit resistance primarily to cationic antimicrobial peptides (CAMPs), such as defensins and cathelicidins, as well as to clinically used peptide antibiotics, particularly daptomycin [90,91].

The presence of the *mprF* gene was confirmed in 22 out of 58 studies (37.93%) [8,12,14,15,16,23,28,30,33,36,38,39,43,44,47,51,60,63,64,65,66,68,69]. For its identification, various bioinformatic tools were applied, with the most frequently used being the BIGSdb-Lm database—10/22 (45.45%) [16,33,36,38,43,64,65,66,88,92] and CARD—10/22 (45.45%) [15,47,51,60,63,64,65,68,69,93]. The other tools included ABRicate—6/22 (27.27%) [14,15,44,48,59,65], and ResFinder—5/22 (22.73%) [15,38,44,51,63]. Less frequently, AMRFinderPlus was used—3/22 (13.64%) [15,39,51], NCBI (BLASTN)—2/22 (9.09%) [44,60], while RGI [93] and ARIBA [47] appeared only once each, in 1 out of 22 studies (4.55%).

The sources of isolation in these studies covered a wide spectrum of samples from food and production environments. The isolates came from three fruit packing plants [14], a meat-processing factory [15], leafy, root, and seed vegetables [16], ready-to-eat meat products [88], broilers [92], raw meat, RTE foods and production environments [33,36,51], fish and seafood products [38], vacuum-packed salmon [43], meat samples and pork-production environments [44], beef products and their preparations [12], cheese [47], cow’s milk [60], dairy farms [63], fruits and berries [65], fish and the fish processing environment [66], bivalves [68], and traditional pork products and their production environment [69].

Other genes associated with peptide antibiotic resistance were the *CdsA* (cardiolipin synthase A) gene [94] and the *PmrF* (phosphoethanolamine transferase) gene [95], detected in only one study (1/58; 1.72%) [60]. The same study also reported an A-Ala-D-Ala fragment, indicating a mechanism of resistance to glycopeptides. The I-Sanger platform, developed by Shanghai Majorbio BioTech Co., Ltd. in Shanghai, China, was used to perform bioinformatics analyses [60].

#### 3.2.5. Sulfonamides Resistance

Genes from the *sul* family encode modified variants of the dihydropteroate synthase (DHPS) enzyme, which plays a key role in folate biosynthesis. Normally, sulfonamides inhibit DHPS activity through competitive bidding, but *sul* gene products have low affinity for sulfonamides while maintaining enzymatic activity, resulting in complete resistance to this group of drugs [96,97].

In the 58 publications analyzed, genes associated with sulfonamide resistance (sul) were detected in 15 cases (15/58; 25.86%) [8,16,18,23,33,34,35,36,37,38,39,40,43,45,66]. Among the bioinformatics tools most commonly used for its detection, BIGSdb-Lm dominated, used in 13 publications (13/15; 86.67%) [8,16,18,33,34,39,40,43,45,60,66,68,88]. Each of the other programs mentioned—ResFinder, ABRicate, CARD, and AMRFinderPlus—were each labeled in one study (1/15; 6.67% each). Specific attributions are as follows: ResFinder [38], ABRicate [66], CARD, and AMRFinderPlus [39].

#### 3.2.6. Tetracycline Resistance

Tetracycline resistance is the most frequently observed antimicrobial resistance trait among acquired antibiotic resistance determinants in *L*. *monocytogenes* isolates, occurring in both clinical and environmental sources, including food-derived strains [83,98,99]. The primary mechanism underlying this resistance involves the acquisition of genes such as *tet*M, *tet*S, and *tet*A, which are commonly associated with mobile genetic elements, particularly conjugative transposons from the Tn916–1545 family and plasmids facilitating horizontal gene transfer across bacterial species [99]. Among these, *tet*M is the most prevalent tetracycline resistance genotype identified in *L*. *monocytogenes* strains. It encodes a ribosomal protection protein [8,44] and has been detected in over 70% of tetracycline-resistant isolates from both environmental and clinical sources, highlighting its dominant role in the spread of resistance [83,99]. Whole genome sequencing enables not only the accurate identification of genes such as *tet*M, *tet*S, and *tet*A, but also provides insight into their genetic context, such as their localization on plasmids, integration within resistance islands, and proximity to other resistance or virulence determinants [51,100]. Our analysis showed that the most commonly used tools for detecting tetracycline resistance genes in *L. monocytogenes* strains were as follows: NCBI BLASTN, CLC Genomics Workbench, BIGSdb-Lm, MEGARes database, ResFinder, CARD, and ARIBA for rapid sequence screening against curated databases of known resistance gene variants.

In the 58 publications analyzed, genes associated with tetracycline resistance were detected in 20 studies (20/58; 34.48%) [8,19,22,24,25,29,30,33,40,41,44,46,47,51,56,59,60,61,67,92]. The most frequently identified gene was *tetM*, present in 13 studies (13/58; 22.41%) [8,19,24,29,30,33,40,44,46,56,59,60,67]. Next in frequency were *tetA* (6/58; 10.34%) [24,28,41,46,51,60], and *tetS* (5/58; 8.62%) [33,44,47,60,67]. *TetR* was reported less frequently (2/58; 3.45%) [22,61]. There were also single or very rare *tet* variants, including *tetC* [51,60], *tetD*, *tetT*, *tet(42)*, and various *tetA* and *tetB* alleles (e.g., *tetA(60)*, *tetA(48)*, *tetB(60)*, *tetB(P)*)—most of these variants were listed in a detailed allele list published by Su et al. (2023) [60]; *tetK* was reported in Shen et al. (2022) [56].

An analysis of the bioinformatic tools used for *tet* gene detection in publications revealed diverse approaches: BIGSdb-Lm was used in 3 out of 20 studies (3/20; 15.0%) [8,28,33], ResFinder (including use via ARIBA/ABRicate) in four studies (4/20; 20.0%) [19,47,59,67], while ABRicate was reported in four studies (4/20; 20.0%) [44,56,59,67]. The CARD program (including RGI/CARD or references to the CARD database within pipelines) appeared in six papers (6/20; 30.0%) [30,41,47,51,60,67]. AMRFinderPlus was identified in two studies (2/20; 10.0%) [51,61], while NCBI BLASTN/NCBI Pathogen Detection appeared in two studies (2/20; 10.0%) [22,44]. CLC Genomics Workbench appeared in one study [24]. The remaining publications used combinations of databases (MEGARes, BacMet, ARIBA, etc.) or described gene aggregate results in a non-standard way (e.g., studies containing extensive lists of allelic variants).

#### 3.2.7. Aminoglycosides Resistance

In the analyzed studies, the *aacA4* gene, associated with resistance to aminoglycosides, was detected in four cases (4/58; 6.90%) [8,33,35,66]. The *aacA4* gene encodes the *aac(6′)-Ib7* enzyme, a key factor in mediating bacterial resistance to aminoglycosides, including tobramycin, kanamycin, and neomycin [101]. This enzyme modifies aminoglycoside molecules by acetylating the amino group at the 6′ position, a change that prevents the antibiotic from binding to the 30S ribosomal subunit. As a result, protein synthesis is disrupted, and the bacteria acquire resistance [102]. Among the bioinformatics tools most commonly used for its detection, BIGSdb-Lm dominated, being used in all cases (4/4; 100.00%) [8,33,35,66]. In addition, Abricate Software was also used in one publication (1/4; 25.00%; [66]). The *aacA4* gene was most frequently detected among *L. monocytogenes* isolates from raw meat and ready-to-eat products [33,35,66].

Other resistance mechanisms have also been reported for aminoglycosides. Only in 1 of the 58 studies analyzed (1/58; 1.72%) were the presence of the *ant(9)-Ia*, *ant(6)-Ia*, and *aph(3′)-IIIa* genes detected, which encode enzymes that modify aminoglycoside molecules and lead to their inactivation. Bioinformatic analyses were performed using the I-Sanger platform (Shanghai Majorbio BioTech Co., Ltd., Shanghai, China) [60].

In a study conducted by Yan et al. (2019) [67], involving the isolation of *L. monocytogenes* strains from various food products in China, the *aadE*, *ant9*, and *aph3* genes were identified, which encode the aminoglycoside O-nucleotidyl transferase enzyme. The presence of these genes indicates a potential mechanism for the resistance of the tested strains to streptomycin. In contrast, Shen et al. (2022) [56] identified, among others, the *aph(4)-Ia* gene, encoding aminoglycoside-inactivating aminoglycoside phosphotransferase, and the *ermC* gene, responsible for 23S rRNA methylation and causing the MLS_B resistance phenotype (resistance to macrolides, lincosamides, and type B streptogramins, including resistance to erythromycin).

#### 3.2.8. Efflux Proteins/Multidrug Resistance

The *mdrL* gene was detected in six cases (6/58; 10.34%) [14,17,27,41,46,48], while the *mepA*, *lde*, and *msrA* genes appeared in three, four, and four publications, respectively, (*mepA*: Choi et al., 2023 [24]; Haubert et al., 2018 [29]; Su et al., 2023 [60]; *lde*: Gelbicova et al., 2019 [27]; Lim et al., 2016 [41]; Matle et al., 2019 [46]; Mota et al., 2020 [48]; *msrA*: Guidi et al., 2023 [28]; Haubert et al., 2018 [29]; Lim et al., 2016 [41]; Matle et al., 2019 [46]).

Among the bioinformatics tools used for detection of these genes, BIGSdb-Lm was actually used only in a minority of the relevant studies (*mdrL*: 1/6, 16.7%—Gelbicova et al., 2019 [27]; *lde*: 1/4, 25.0%—Gelbicova et al., 2019 [27]; *msrA*: 1/4, 25.0%—Guidi et al., 2023 [28]). ABRicate was used in two of the *mdrL*-positive studies (2/6, 33.3%—Méndez Acevedo et al., 2024 [14]; Mota et al., 2020 [48]). The CARD (or CARD-based RID/AMR callers) appears in single *mdrL*-positive studies (1/6, 16.7%—Lim et al., 2016 [41]) and was also the tool reported for detecting *mepA* in Su et al., 2023 [60] (*mepA*: 1/3, 33.3%). Other databases/tools reported across these papers include MEGARes, BacMet, Bionumerics, and the CLC Genomics Workbench.

#### 3.2.9. Other Genes Detected in the Analyzed Studies

In the analyzed publications, the *ACC-3* gene (encoding aminoglycoside-modifying enzymes [101]) was reported in only one study (1/58; 1.72%) [60]. In addition, the SRT-1 gene, which is also responsible for resistance to this class of antibiotics, was detected. In addition, the same study detected the presence of the *Erm(34)*, *ErmH*, and *ErmB* genes, which encode rRNA methyltransferases [103]. The *ErmB* gene in particular was identified in most strains exhibiting phenotypic resistance to erythromycin, as confirmed, among others, in the NDDQ-065-1 strain described by Su et al. (2023) [60]. In addition, the presence of the *LpeA* gene, which also determines resistance to macrolides, was detected. The study also identified genes conferring resistance to other classes of antibiotics. These include the *TaeA* gene, which encodes resistance to pleuromutilins [104], and the *Vatl* gene, which confers resistance to streptogramins [105]. Bioinformatic analyses were performed using the I-Sanger platform (Shanghai Majorbio BioTech Co., Ltd., Shanghai, China) [60].

A study by Yan et al. (2019) [67] showed that *L. monocytogenes* isolates exhibit multidrug resistance (MDR) and contain the *cat* gene, which confers resistance to chloramphenicol, and the *erm(B)* gene, which encodes resistance to macrolide antibiotics. In this study, the chloramphenicol resistance marker, cat, was consistently identified in isolates positive for the *tet(S)* gene, indicating possible co-occurrence and links between resistance determinants in the *L. monocytogenes* genome [67]. An analysis conducted by Brown et al. (2024) [22], covering *L. monocytogenes* strains isolated from both dairy products and the environment of food-processing plants, revealed the presence of, among others, the *emrE* and *emrC* genes. These genes are responsible for encoding efflux pumps belonging to the MFS (Major Facilitator Superfamily), which participate in the active removal of antimicrobial compounds from the cell, thereby contributing to the increased tolerance of bacteria to antibiotics and biocides. The detection of these resistance determinants was confirmed using the NCBI BLASTN tool [22].

In the genome of *L. monocytogenes* food isolates (in dairy products), the TUFAB_7 gene, associated with resistance to efamycin, was also detected [47]. However, analysis using different databases revealed discrepancies—the gene was not identified in the CARD database, but its presence was confirmed using the MEGARes database [47].

A study by Su et al. (2023) [60] demonstrated the presence of the *abeS* gene, associated with resistance to macrolides through an efflux mechanism, which reduces the effectiveness of these antibiotics [106]. The same study also identified other resistance genes (*arlR*, *bcrA*, *dfrG*, *fdP*, *kdpE*, *liaR*, *liaS*, *lmrC*, *lmrD*, *lnuB*, *IsaE*, *mdtL*, *patB*, *rphB*, *rpoB*, and *folp*), which determine, among other things, the modification of the target site of action of drugs, the activation of efflux pumps, and the regulation of the response to antibiotic stress. Importantly, the *lmrB* gene, also associated with macrolide resistance, was previously described by Lim et al. (2016) [41], confirming its wider prevalence and role in the development of multidrug resistance. Such a wide repertoire of determinants highlights the high potential of the studied strains to develop multidrug resistance, posing a serious challenge for antimicrobial therapies [41,60].

Choi et al. (2023) [24] described *L. monocytogenes* isolates from fungi in which numerous genes associated with β-lactam antibiotic resistance were found, such as *pbp*, *pbpX*, *pbpH*, *ponA*, *pbpB*, and *pbpF*. In addition, genes associated with clindamycin resistance (*emrY*, *emrB*) were identified, as well as multidrug resistance determinants, including *mdrL* and *norM*, responsible for the action of efflux pumps. The results of the genomic analysis suggest that the *L. monocytogenes* SMFM2019-FV16 strain may be resistant to penicillin, tetracycline, and clindamycin.

Hauber et al. (2018) [29] highlighted the importance of single mutations in shaping the resistance profile of *L. monocytogenes*. It has been shown that changes in the *efTu* gene (C4881_02615) lead to bacterial resistance to elfamycin. Importantly, a comparative analysis showed that the reference genome of the *L. monocytogenes* EGD-e strain, which is used as a reference in numerous studies, lacks three gene-encoding efflux pumps identified in other isolates. This means that their presence is not a universal feature of the entire species but rather indicates the adaptive differentiation of individual strains in response to environmental and antibiotic stress [29].

Matle et al. (2019) [46] showed that all *L. monocytogenes* isolates in this study contained the *mecC* gene, which encodes a mechanism of resistance to β-lactam antibiotics [107]—the primary group of drugs used to treat listeriosis in humans, often in combination with aminoglycosides. In addition, the *lmrB* gene, responsible for resistance to lincomycin through an efflux pump mechanism, was detected. The presence of these genes indicates that *L. monocytogenes* possesses both genetic determinants of resistance to therapeutically critical antibiotics and mechanisms that enable tolerance to other antimicrobial agents. This accumulation of resistance genes highlights the potential clinical threat and the importance of genomic monitoring of environmental and food strains for their resistance profile [46].

Ji et al. (2023) [33] identified ten genes responsible for antibiotic resistance, including *lmo0441* (penicillin-binding protein—PBP-like; associated with resistance to cephalosporins) [33]. Similar observations were reported by Kragh et al. (2024) [34], who detected the *lmo0441* gene in all analyzed isolates, indicating its role in determining resistance to cephalosporins. Similarly, Astoshkin et al. (2021) [20] confirmed the presence of the *lmo0441* gene, proving its widespread occurrence in various populations of *L. monocytogenes*. These results indicate that *lmo0441* may be a key determinant of cephalosporin resistance within the species [20].

## 4. Epidemiological and Health Consequences of Detected Resistance Patterns

The detection of resistance genes in *L. monocytogenes* using NGS methods is an important element of contemporary epidemiological research and public health surveillance. The results of genomic analyses reveal both the scale of the spread of resistant strains in the food environment and the potential clinical consequences for patients infected with this microorganism [83,108]. Research confirms that resistance genes are often transferred by plasmids and mobile elements, which promotes their rapid spread among both environmental bacteria and clinical pathogens [98,109]. From a clinical perspective, ampicillin or penicillin in combination with gentamicin remains the standard treatment, but reported cases of aminoglycoside resistance limit the effectiveness of treatment [110]. Infections caused by resistant strains may be more severe, with a higher risk of recurrence and higher mortality, especially among immunocompromised patients, pregnant women, and newborns [111].

At the population level, the detection of resistance patterns in *L. monocytogenes* indicates growing challenges in controlling this bacterium throughout the food chain. Strains resistant to tetracyclines, macrolides, and fluoroquinolones are increasingly isolated in food-production environments, which increases the risk of their transmission to consumers [112]. Geographical diversity is also significant. Regions with the intensive use of antibiotics in animal husbandry have a higher incidence of antibiotic resistance [113]. Furthermore, resistant strains can persist for long periods in food-production facilities, increasing their epidemic potential. NGS not only enables the identification of resistance patterns but also allows the precise linking of clinical infection sources to specific production lines, which significantly supports preventive measures [114].

## 5. Conclusions and Limitations of the Research

Whole-genome sequencing (WGS) is an effective tool for detecting both innate and acquired resistance determinants in *L. monocytogenes* originating from food and processing environments. The studies analyzed showed that the *fosX* gene is virtually ubiquitous as part of the core genome, while other resistance genes show variable frequencies depending on the sample type and region of origin of the strain. Phenotype prediction based on sequence is high in most cases, but the observed discrepancies highlight the need to supplement genotypic analyses with phenotypic testing before making clinical or regulatory decisions.

A review of the literature identifies several key methodological limitations. The most frequently highlighted problem is the heterogeneity of data reporting—many studies do not provide complete phenotypic results, which prevents direct verification of the accuracy of genotype–phenotype predictions. Differences in the sequencing platforms used and depth of coverage affect data quality and may lead to the overlooking of less-common resistance determinants, especially those located on mobile genetic elements. Furthermore, the mere presence of a gene in the genome does not necessarily correlate with the phenotype–gene expression and regulation, as well as point mutations in target genes (e.g., penicillin-binding proteins) that can significantly affect antibiotic susceptibility, and standard bioinformatics procedures often do not take this into account. Models combining genotype and phenotype data are also prone to systematic errors resulting from unrepresentative samples or poor generalization between bacterial populations. The lack of standardized reporting protocols and unified databases limits the comparability of studies.

In view of the above, it is recommended to implement uniform reporting standards (including the mandatory sharing of phenotypic results), develop and validate consistent bioinformatics tools, and build extensive, consolidated databases. Where possible, long-read sequencing or hybrid approaches should be used to better determine the genomic context of resistance determinants. Future research should focus on studying the expression and regulatory mechanisms of common resistance loci, explaining the reasons for genotype–phenotype discordance, and evaluating the practical aspects of implementing NGS in food safety systems.

## Figures and Tables

**Table 1:** The characterization and antibiotic resistance profile of sequenced *L. monocytogenes* strains from food and its production environment.

No	Number of Sequenced Strains	Serotype/Serogroup	Source of Samples	Sequencing Method	Genotypic Antibiotic Resistance		Phenotypic Antibiotic Resistance		Reference
					Method (Program)	Antibiotic Resistance Genes	Method	Phenotype of Resistance	
**1.**	45	NA	Three tree fruit packing facilities	Illumina MiSeq (San Diego, CA, USA) (600 cycles, 300 bp paired-end reads)	ABRicate (v1.0.1) in GalaxyTrakr, using the MEGARes (v3.0)	*fosX*, *lin*, *mdrL*, *mprF*, *norB*	NA	NA	[14]
**2.**	18	1/2a, 1/2c	Meat-processing facility	Illumina HiSeq 1500 (San Diego, CA, USA) (300 cycles, 150 bp paired-end reads)	ABRicate (v.0.9.9), CARD, AMRFinderPlus, ResFinder)	*norB*, *mprF* (CARD), *lin* (ARG-ANNOT and CARD), *fosX* (ResFinder and NCBI AMRFinderPlus)	NA	NA	[15]
**3.**	12	1/2a, 4b	Leafy greens, root vegetables, and seeded vegetables	Illumina X10 (San Diego, CA, USA) (300 cycles, 150 bp paired-end reads)	BIGSdb-Lm database	*fosX*, *lin*, *mprF*, *norB*, *sul*	NA	NA	[16]
**4.**	54	1/2a, 3a,1/2b, 3b, 7	Cheese and cheese-processing surfaces	Novogene Genomics Service (Novogene Co., Ltd., Cambridge, UK)	Bionumerics version 8.1.1 (bioMérieux, Marcy-l’Étoile, France)	*fosX*, *mdrL*, *mdrM*	Disk diffusion method, E-test	Ciprofloxacin (intermediately resistant)	[17]
**5.**	21	IIa, IIb, IIc, IVb, L	Food	Illumina NovaSeq 6000 (San Diego, CA, USA) (300 cycles, 150 bp paired-end reads)	BIGSdb-Lm	*fosX*, *lin*, *norB*, *sul*	NA	NA	[18]
**6.**	21	4bV, 4b/4d/4e	Retail deli environments	Illumina MiSeq (San Diego, CA, USA)	ResFinder (v.3.2)	*tetM*, *fosX*	NA	NA	[19]
**7.**	9	1/2a–3a, 1/2b–3b-7, 1/2c–3c	Beef, fish, and poultry	Illumina MiSeq (San Diego, CA, USA)	BIGSdb-Lm database	*fosX*, *lmo0441*, *lin*, *norB*, *lmo0224*	Microdilution method	Tetracyclines, macrolides, sulfonamides, beta-lactams.	[20]
**8.**	3	NA	Resh-cut fruits and vegetables	Illumina HiSeq (San Diego, CA, USA)	ResFinder	No resistance genes detected	VITEK 2 AST- GN05/GP67 Test Kit (bioM ’erieux Corporate)	No resistant strains	[21]
**9.**	54	NA	Dairy processing plants	Illumina HiSeq 1500 (San Diego, CA, USA) (300 cycles, 150 bp paired-end reads)	NCBI BLASTN	*tetR*, *emrE*, *emrC*	NA	NA	[22]
**10.**	18	NA	Rady-to-eat meat products	Illumina NextSeq 500 (San Diego, CA, USA) (300 cycles, 150 bp paired-end reads)	BIGSdb-Lm database	*fosX*, *mprF*, *norB*, *sul*	Microdilution method with the Sensititre OptiRead Automated Fluorometric Plate Reading System (Thermo Scientific, Monza, Italy)	Cephalosporins, penicillin, tetracycline, trimethoprim/sulfamethoxazole	[23]
**11.**	7	1/2a-3a, 1/2b-3b	Enoki mushrooms	PacBio RS II platform (Pacific Biosciences, Menlo Park, CA, USA)	CLC Genomics Workbench version 12.0 (Qiagen)	*pbp*, *pbpX*, *pbpH*, *ponA*, *pbpB*, *pbpF*, *tetA*, *tet*(*M*), *emrY*, *emrB*, *emrB*, *norB*, *norm*, *mepA*, *mdtL*	Disk diffusion method	Penicillin G, ampicillin, tetracycline, clindamycin.	[24]
**12.**	4	1/2c, 4b	Food	Illumina MiSeq (San Diego, CA, USA) (600 cycles, 300 bp paired-end reads)	Mauve, Artemis, and BRIG (BLAST Ring Image Generator)	A number of non-specific multidrug efflux genes, as well as genes with putative functions in increasing resistance of the bacterium to lincomycin, quinolone, β-lactams, and tetracycline	NA	NA	[25]
**13.**	60	IVb, IVb, IIa	Cattle farms, cattle abattoirs, and retail outlets	Illumina MiSeq (San Diego, CA, USA) (500 cycles, 250 bp paired-end reads)	ABRicate	*fosX*, *vga(G)*	NA	NA	[26]
**14.**	4	1/2a	Rabbit meat	Illumina HiSeq (San Diego, CA, USA) (300 cycles, 150 bp paired-end reads) Illumina MiSeq (San Diego, CA, USA) (600 cycles, 300 bp paired-end reads)	BIGSdb-Lm database at Institute Pasteur	*mdrL*, *lde*	NA	NA	[27]
**15.**	122	NA	Broiler chickens	Illumina NextSeq 500 (San Diego, CA, USA) (300 cycles, 150 bp paired-end reads)	BIGSdb-Lm	*norB*, *mprF*, *lin*, *fosX*, *parC*, *msrA*, *tetA*	NA	NA	[28]
**16.**	1	1/2a	Fresh mixed sausage	Illumina MiSeq (San Diego, CA, USA) (500 cycles, 250 bp paired-end reads)	MEGARes database	*tetM*, *mepA*, *msrA*, *norB*, *efTu*, *gyrA*	Disk diffusion method	Streptomycin, erythromycin, clindamycin, rifampicin, meropenem, trimethoprim–sulfamethoxazole, tetracycline	[29]
**17.**	19	NA	Foods	Illumina MiSeq (San Diego, CA, USA) (500 cycles, 250 bp paired-end reads)	Resistance gene identifier (RGI) software (v.5.1.1), CARD (v.3.1.1)	*fosX*, *lin*, *mprF*, *tet(M);* (*tet(M)* is not known to be among the isolates from food)	NA	NA	[30]
**18.**	20	NA	Produce, nuts, and spices	Illumina MiSeq (San Diego, CA, USA) (500 cycles, 250 bp paired-end reads)	NCBI AMRFinder process in the NCBI Pathogen detection system, 4 based on the Bacterial Antimicrobial Resistance Reference Gene Database.	*fosX*, *lin*, *abc-f*	NA	NA	[31]
**19.**	100	IIa, IIb, IIc, IVb, IVb-v1	Meat and vegetable processing facilities	Illumina HiSeq (San Diego, CA, USA) (500 cycles, 250 bp paired-end reads)	BLAST+ (v.2.9.0), ResFinder (v.3.1.0)	*fosX*	NA	NA	[32]
**20.**	322	Ia, IIb, IIc, IVb and L	Raw meat, raw poultry, ready-to-eat food, aquatic products, and unknown food sources	Illumina HiSeq X PE150 (San Diego, CA, USA) (coverage rate of more than 100-fold)	BIGSdb-Lm database	*fosX*, *lmo0441*, *lin*, *norB*, *mprF*, *sul*, *aacA4*, *tetS*, *tetM*	NA	NA	[33]
**21.**	240	1/2a, 1/2b, 4b, 1/2c	Foods, food-processing environments	Illumina NextSeq (San Diego, CA, USA) (300 cycles, 150 bp paired-end reads)	BIGSdb-Lm database at Institute Pasteur	*fosX*, *lin*, *norB*, *lmo0441*, *sul*	NA	NA	[34]
**22.**	48	IIb, IVb	Ready-to-eat food of animal origin (e.g., ham, sausages, or meat), and food-processing environments	Illumina MiSeq (San Diego, CA, USA), Illumina NextSeq500 (San Diego, CA, USA)	BIGSdb-Lm database	*fosX*, *lin*, *norB*, *sul*, *aacA4*	NA	NA	[35]
**23.**	91	NA	Raw meat, ready-to-eat food and food-production environments	Illumina MiSeq (San Diego, CA, USA) (50× average coverage)	BIGSdb-Lm	*fosX*, *lin*, *norB*, *sul*, *mprF*	NA	NA	[36]
**24.**	44	1/2a-3a, 1/2b-3b-7, 4b-4d-4e	Casing soil, mushrooms, equipment, and frozen mushrooms	Illumina Miseq (San Diego, CA, USA) (300 cycles, 150 bp paired-end reads)	BIGSdb-Lm database	*fosX*, *sul*, *norB*, *lin*	Disk diffusion method	No resistant strains	[37]
**25.**	38	1/2a, 1/2b, 4b	Seafood products and other food	CosmosID (Germantown, MD, USA), Inqaba Biotechnical Industries (Pretoria, South Africa)	ResFinder (v.4.1), BIGSdb-Lm	*fosX*, *lin*, *mprF*, *norB*, *sul*	Disk diffusion method	Tetracycline, sulphamethoxazole/trimethoprim, erythromycin, chloramphenicol	[38]
**26.**	1	IIb	Pork	Illumina NextSeq P1 600 cycles (San Diego, CA, USA) (600 cycles, 300 bp paired-end reads)	AMRFinderPlus (v.3.11.26), BIGSdb_LM	*fosX*, *vga(G)*, *mprF*, *norB*, *sul*	NA	NA	[39]
**27.**	25	1/2b, 1/2a, 1/2c, 4b	Foods	Illumina MiSeq (San Diego, CA, USA) (300 cycles, 150 bp paired-end reads), Oxford Nanopore sequencing technologies (Oxford Nanopore Technologies, Oxford, UK)	*Listeria* MLST database	*fosX*, *norB*, *sul*, *lin*, *tetM*	Disk diffusion method	Lincomycin, penicillin G	[40]
**28.**	2	4b	Fried fish and salad	Illumina HiSeq 2000 (San Diego, CA, USA)	Resistance Gene Identifier (RID) of the CARD	*tetA*, *lmrB*, *fosX*, *msrA*, *lde*, *mdrL*	NA	NA	[41]
**29.**	48	NA	Ready-to-eat meat products	Illumina HiSeq 1500 (San Diego, CA, USA) (500 cycles, 250 bp paired-end reads)	ABRicate version (v.0.8)	*lin*, *fosX*,	NA	NA	[42]
**30.**	1	IIa	Vacuum-packaged sliced salted salmon products	Illumina MiSeq (San Diego, CA, USA) (600 cycles)	BIGSdb database	*fosX*, *lin*, *mprF*, *norB*, *sul*	NA	NA	[43]
**31.**	152	IIa, IIb, IIc, IVb,	Raw meat, processed meat, ready-to-eat meat products and environmental samples collected from a commercial pig farm environment	Illumina HiSeq and MiSeq (Illumina, San Diego, CA, USA)	ABRicate and NCBI	*fosX*, *lin*, *norB*, *mprF*, *tetM*, *tetS*	NA	NA	[44]
**32.**	24	IIa, IVb, IIb, IIc	Beef and beef-based products	Illumina MiSeq (Illumina, San Diego, CA, USA)	ResFinder, CARD, and NCBI	*lin*, *norB*, *fosX*, *mprF*	NA	NA	[12]
**33.**	35	IIa, IVb, IIc, IIb,	Corn, meat, vegetables, pepper, fish, pineapple, salad, easter sausage, smoked salmon, salami, barbecue chorizo, cooked peeled prawn, smoked sardine, smoked fish product, surface sponge, sweet chorizo, matured cheese, surface sponge, blood sausage, Majorcan sausage, and chorizo	Illumina NextSeq 500 (San Diego, CA, USA)	BIGSdb database	*fosX*, *lin*, *norB*, *sul*	Disk diffusion method, Microdilution method	No resistant strains	[45]
**34.**	6	2a, 4b	Meat and meat products	Illumina MiSeq (San Diego, CA, USA) (600 cycles, 300 bp paired-end reads)	BacMet, MEGARes and nonredundant antibiotic resistance database (noradab)	*fosX*, *tetA*, *tetM*, *mecC*, *mrB*, *msrA*, *lde*, *mdrL.*	NA	NA	[46]
**35.**	45	NA	Cheese	Illumina NextSeq (San Diego, CA, USA) (300 cycles, 150 bp paired-end reads)	ARIBA software (v.2.11.1) (CARD, MEGAres, and ResFinder databases)	*fosX*, *mprF*, *norB* (CARD), *Gyra_23*, *TUFAB-7* (MEGAres), *tetS* (Resfinder)	Microdilution technique, using panels of lyophilized antibiotics for Gram-positive bacteria (Sensititre 36GPALL1F, ThermoScientific®, Waltham, MA, USA)	No resistant strains	[47]
**36.**	21	1/2a, 1/2b, 4b	Frozen food, ready-to-eat food, deli meat, and cheese.	Illumina MiSeq (San Diego, CA, USA)	ABRicate software (ResFinder, CARD, NCBI, AMRFinderPlus, MEGARes BLAST tool)	*fosX*, *lin*, *norB*, *lde*, *mdrL*, *fepA*	Disk diffusion method	Ciprofloxacin	[48]
**37.**	2431	NA	Food	Illumina NextSeq 500 (San Diego, CA, USA) (300 cycles, 150 bp paired-end reads)	BIGSdb-Lm	*fosX*, *norB*, *lin*, *sul*, *pbp*, *tetM*, *ermB*, *mphB*, *fexA*, *dfrD*, *InuG*, *aacA4*, *aphA*, *dfrD*	NA	NA	[8]
**38.**	2	NA	Mung bean sprouts	Illumina iSeq100 (San Diego, CA, USA) (300 cycles, 150 bp paired-end reads)	ResFinder, AMRonize, AMRFinder, ABRicate, Staramr	*fosX*, *lin*	NA	NA	[49]
**39.**	54	1/2a	Processing plants	Illumina NextSeq 500 (San Diego, CA, USA) (300 cycles, 150 bp paired-end reads), Illumina MiSeq (San Diego, CA, USA) (500 cycles, 250 bp paired-end reads)	NCBI Pathogen Detection Pipeline	*fosX*, *lin*	NA	NA	[50]
**40.**	14	1/2a, 1/2b	Cheeses, cooked meats (artisanal ham, pâté, sausages, and blood sausage), pre-processed fruits and vegetables (chopped fruit, fruit salads with strawberries, melon, peaches, and leafy vegetable salads), and meals and mixed dishes with raw and/or cooked ingredients	Illumina MiSeq (San Diego, CA, USA) (600 cycles, 300 bp paired-end reads)	CARD, AMRFinderPlus (v.3.2.3) database	*fosX*, *lin*, *norB*, *mprF*, *tetA*, *tetC*	Disk diffusion method	Ampicillin	[51]
**41.**	54	4b, 1/2b, 4b, 1,2a, 1/2c, 4b (4b-v1)	Turkey processing plants	Illumina HiSeq (San Diego, CA, USA) (500 cycles, 250 bp paired-end reads)	NCBI Pathogen Detection Pipeline	*fosX*, *lin*, *abc-F*	NA	NA	[52]
**42.**	160	IIc, IIb, IIa, IVb	Raw milk and fresh meat	Illumina NextSeq2000 (San Diego, CA, USA) (300 cycles, 150 bp paired-end reads)	NCBI AMRFinder+ (v.3.11.2)	*fosX*, *lin*	NA	NA	[53]
**43.**	1	4b	Slaughterhouse	Illumina Novaseq (San Diego, CA, USA) (300 cycles, 150 bp paired-end reads)	CARD	*pgs*, *arlR*, *bcrA*, *rphA*, *rpoB*, *vanR*, *vanG*, *mdtG*, *lmrC*, *lmrD*, *norB*, *adeC*, *FosX*	Disk diffusion method	Polymyxin, ceftazidime	[54]
**44.**	93	NA	Food-production plants	Illumina MiSeq (San Diego, CA, USA) (602 cycles, 301 bp paired-end reads)	ResFinder (v.3.0)	*fosX*	Commercial test system Micronaut S *Listeria* MHK-2 (Merlin Gesellschaft für Mikrobiologische Diagnostika mbH, Bornheim, Germany),	Daptomycin, tigecycline, meropenem, ciprofloxacin (susceptible, increased exposure [I]), and rifampin (I).	[55]
**45.**	89	1/2a, 3a, 1/2c, 3c	Frozen beef, frozen pork, fresh fish, fresh aquatic products except fish, frozen chicken, frozen sheep casing, and dairy food products	Illumina Hiseq×10 (San Diego, CA, USA) (300 cycles, 150 bp paired-end reads)	ABRicate software	*aph(4)Ia*, *ermC*, *fexA*, *tetK*, *tetM*	Microdilution method	Oxacillin, daptomycin, chloramphenicol, tetracycline, ciprofloxacin, erythromycin, imipenem, clindamycin, ciprofloxacin (Intermediate levels of resistance), chloramphenicol (Intermediate levels of resistance)	[56]
**46.**	13	NA	Food products or food-processing environments	Illumina NextSeq 2000 (San Diego, CA, USA) (300 cycles, 150 bp paired-end reads)	ResFinder (v.2.0), CARD	*fosX lin*, *norB*, *tetM*	Disk diffusion method	Trimethoprim–sulfamethoxazole, erythromycin, penicillin	[57]
**47.**	15	NA	Spinach, environmental swab (drain), red leaf lettuce, beetroot, pea shoots, and baby salad kale	Illumina HiSeq (San Diego, CA, USA) (500 cycles, 250 bp paired-end reads)	ABRicate (v.0.8) (BLAST+ & EMBOSS, Resfinder)	*fosX*, *lin*	NA	NA	[58]
**48.**	142	NA	Food	Illumina Hiseq PE150 by Novogene (Beijing, China)	ABRicate (v.1.0.0) pipeline by the Resfinder database	*fosX*, *tetM*	NA	NA	[59]
**49.**	8	NA	Bovine milk	Illumina NovaSeq 6000 (San Diego, CA, USA) (300 cycles, 150 bp paired-end reads)	CARD	*ANT(9)-Ia*, *ANT(6)-Ia*, *APH(3′)-IIIa*, *SRT-1*, *ACC-3*, *mecA*, *PBP2x*, *PBP1a*, *dfrG*, *FosX*, *fusA*, *fusB*, *fusE*, *vanHF*, *vanHD*, *vanRM*, *vanTE*, *vanYM*, *vanRG*, *vanSM*, *vanRE*, *vanTG*, *vanRF*, *vanRI*, *norB*, *patB*, *mdtG*, *mepA*, *lmrB*, *lmrC*, *lmrD*, *lsaA*, *lsaE*, *salA*, *macB*, *LpeA*, *oleC*, *abeS*, *efrA*, *msrC*, *bcrA*, *cmrA*, *fexA*, *vgaE*, *vgaALC*, *optrA*, *ErmB*, *ErmH*, *Erm(34)*, *cfrA*, *lnuB*, *vatI*, *vatB*, *sul4*, *folP*, *tetA(60)*, *tetA(48)*, *tetA(46)*, *tetT*, *tetC*, *tetD*, *tetM*, *tetS*, *tet(42)*, *tetB(60)*, *tetB(P)*, *tcr3*, *PmrF*, *mprF*, *liaR*, *liaS*, *cls*	Microdilution method	Penicillin, tetracycline, trimethoprim-sulfamethoxazole, erythromycin	[60]
**50.**	85	NA	Ready-to-eat fish and meat	Illumina MiSeq (San Diego, CA, USA) (500 cycles, 250 bp paired-end reads)	AMRFinderPlus (v.3.10.14)	*fosX*, *lin*, *Tn6188*, *tetR*	NA	NA	[61]
**51.**	16	1/2a, 1/2b, 3a, 4a, 4b, 4c, 4e	Food and food-related environments.	Illumina NextSeq500 (San Diego, CA, USA) (300 cycles, 150 bp paired-end reads)	ResFinder	*fosX*	NA	NA	[62]
**52.**	45	IVb, IIa, IIb	Dairy cattle farms forage, water, raw tank milk, the tank milk filters, fresh feces, stored manure, and soil	Illumina MiSeq (San Diego, CA, USA) (300 cycles, 150 bp paired-end reads)	Resfinder, CARD, ARG-ANNOT databases by using ABRicate	*fosX*, *lin*, *norB*, *mprF*	Microdilution method	Ampicillin, ciprofloxacin, erythromycin, tetracycline, vancomycin, meropenem	[63]
**53.**	16	NA	Food	Illumina MiSeq and NextSeq 500/550 (San Diego, CA, USA)	BIGSdb-Lm database, CARD	*fosX*, *lin*, *mprF*, *norB*	NA	NA	[64]
**54.**	8	IVb, IIa	Primary production and processing companies’ fresh fruit and frozen berries	Illumina NextSeq 550 (San Diego, CA, USA) (300 cycles, 150 bp paired-end reads)	ABRicate (v.1.0.1), CARD	*fosX*, *mprF*, *lin*, *norB*	BD Phoenix System (Becton Dickinson, Franklin Lakes, NJ, USA)	Penicillin, fosfomycin, ciprofloxacin, moxifloxacin	[65]
**55.**	28	IIa, IVb	Raw fish material, i.e., fresh and frozen Atlantic salmon (*Salmo salar*), swabs from fish-production environments (n = 200) as well as samples from ready-to-eat (RTE) fish products, i.e., cold-smoked Atlantic salmon, marinated (gravlax) Atlantic salmon, and cold-smoked rainbow trout (*Oncorhynchus mykiss*)	Illumina MiSeq (San Diego, CA, USA) (600 cycles, 300 bp paired-end reads)	BIGSdb-Lm, ABRicate software	*fosX*, *lin*, *mprF*, *norB*, *sul*, *aacA4*	NA	NA	[66]
**56.**	28	IIa, IIb, IIc, IVb	National food surveillance	Novogene (Beijing, China) on an Illumina HiSeq (San Diego, CA, USA)	ABRicate software package (ResFinder v.2.1.37, ARG-ANNOT v.438, CARD v.2.0.339 databases)	*aadE*, *ant9*, *aph3*, *cat*, *erm(B)*, *lsaE*, *lnuB*, *drfG*, *tet(S)*, *tet(M)*	Microdilution method	tetracycline, erythromycin, chloramphenicol, trimethoprim/sulfamethoxazole	[67]
**57.**	30	NA	Bivalves	Illumina MiSeq (San Diego, CA, USA) (600 cycles, 300 bp paired-end reads)	CARD	*norB*, *lin*, *mprF*, *fosX*, *sul*	Microdilution method	Fosfomycin, lincomycin, tetracycline	[68]
**58.**	20	IIa, IVb	Traditional pork products, including drycured ham (Njeguški pršut), pork tenderloin, pancetta (thin, dry bacon), sausages, and processing environment (swabs from surfaces and drains)	Illumina MiSeq NexteraXT (San Diego, CA, USA) (600 cycles, 300 bp paired-end reads)	CARD	*mprF*, *fosX*	NA	NA	[69]

NA—not applicable.

## Data Availability

The original contributions presented in this study are included in the article and Supplementary Material. Further inquiries can be directed to the corresponding author.

## Supplementary Materials

The following supporting information can be downloaded at: https://www.mdpi.com/article/10.3390/ijms262010112/s1.
